# Adverse Social Experiences in Adolescent Rats Result in Enduring Effects on Social Competence, Pain Sensitivity and Endocannabinoid Signaling

**DOI:** 10.3389/fnbeh.2016.00203

**Published:** 2016-10-20

**Authors:** Peggy Schneider, Laura Bindila, Christian Schmahl, Martin Bohus, Andreas Meyer-Lindenberg, Beat Lutz, Rainer Spanagel, Miriam Schneider

**Affiliations:** ^1^Research Group Developmental Neuropsychopharmacology, Institute of Psychopharmacology, Central Institute of Mental Health, Medical Faculty Mannheim, University of HeidelbergMannheim, Germany; ^2^Institute of Psychopharmacology, Central Institute of Mental Health, Medical Faculty Mannheim, University of HeidelbergMannheim, Germany; ^3^Institute of Physiological Chemistry, University Medical Center of the Johannes Gutenberg University MainzMainz, Germany; ^4^Department of Psychosomatic Medicine and Psychotherapy, Central Institute of Mental Health, Medical Faculty Mannheim, University of HeidelbergMannheim, Germany; ^5^Institute for Psychiatric and Psychosomatic Psychotherapy, Central Institute of Mental Health, Medical Faculty Mannheim, University of HeidelbergMannheim, Germany; ^6^Faculty of Health, University of AntwerpAntwerp, Belgium; ^7^Department of Psychiatry and Psychotherapy, Central Institute of Mental Health, Medical Faculty Mannheim, University of HeidelbergMannheim, Germany

**Keywords:** peer-rejection, social play, social behavior, endocannabinoid system, CB1 receptor, female rats, adolescence, adverse experience

## Abstract

Social affiliation is essential for many species and gains significant importance during adolescence. Disturbances in social affiliation, in particular social rejection experiences during adolescence, affect an individual’s well-being and are involved in the emergence of psychiatric disorders. The underlying mechanisms are still unknown, partly because of a lack of valid animal models. By using a novel animal model for social peer-rejection, which compromises adolescent rats in their ability to appropriately engage in playful activities, here we report on persistent impairments in social behavior and dysregulations in the endocannabinoid (eCB) system. From postnatal day (pd) 21 to pd 50 adolescent female Wistar rats were either reared with same-strain partners (control) or within a group of Fischer 344 rats (inadequate social rearing, ISR), previously shown to serve as inadequate play partners for the Wistar strain. Adult ISR animals showed pronounced deficits in social interaction, social memory, processing of socially transmitted information, and decreased pain sensitivity. Molecular analysis revealed increased CB1 receptor (CB1R) protein levels and CP55, 940 stimulated [^35^S]GTPγS binding activity specifically in the amygdala and thalamus in previously peer-rejected rats. Along with these changes, increased levels of the eCB anandamide (AEA) and a corresponding decrease of its degrading enzyme fatty acid amide hydrolase (FAAH) were seen in the amygdala. Our data indicate lasting consequences in social behavior and pain sensitivity following peer-rejection in adolescent female rats. These behavioral impairments are accompanied by persistent alterations in CB1R signaling. Finally, we provide a novel translational approach to characterize neurobiological processes underlying social peer-rejection in adolescence.

## Introduction

The need for stable social attachments represents a ubiquitous phenomenon within social groups. Social affiliation is a fundamental requirement that provides security and mental health and thus, affiliative and reciprocal sociality is observable across species (Williams, 2006). Social rejection, the experience of being excluded or ostracized, can be detected in almost all social species and has been observed in humans across cultures and various developmental stages (Williams, 2006). Rejection by group members is only one form of negative social interaction, but since it threatens the need for social inclusion, and thus survival, individuals are more sensitive to rejection as compared to other types of aversive social interaction (Goodall, 1986). In humans, experience of rejection has been reported to interfere with pain processing (Bernstein and Claypool, 2012) and is involved in the development of neuropsychiatric disorders such as depression, borderline personality disorder (BPD) and anxiety disorders (Hawthorne, 2008; Stanley and Siever, 2010).

Adequate peer-contact gains particular significance during adolescence. From an evolutionary perspective, adolescence is crucial for the maturation of social skills, which are essential for successful social inclusion and reproduction and enable an individual to slowly separate from the protective influence of the family (Spear, 2000). This individuation from parents is accompanied by a deepening of relationships and intimacy with peers, especially among girls (Larson and Richards, 1991). Teenagers spend more time interacting with peers than during any other developmental period and are more susceptible to peer-influence. Sensitivity towards social rejection has been reported to be higher during adolescence than in adulthood, with girls being particularly sensitive to the perception of peer-rejection (Sebastian et al., 2010). Adolescent social rejection elicits strong feelings of distress, has been found to negatively affect psychological well-being and can result in severe adverse health outcomes (Lev-Wiesel et al., 2006), whereas adequate social support has been linked to positive effects on mental health (Kawachi and Berkman, 2001). Surprisingly, the neurobiological mechanisms mediating the consequences of social rejection remain largely unknown. At this point valid animal models represent a crucial link to increase our insight into mechanistic processes underlying social rejection and its consequences.

We previously reported on a unique experimental approach to model social rejection in rats, by modulating the ability of adolescent female animals to engage in social play behavior either in acute social encounters or over longer periods throughout adolescence (Schneider et al., 2014, 2016). Peer-directed playful activities have a considerable incentive value during adolescence and are crucial for the development of social competence (Trezza et al., 2010). Furthermore, previous studies found that depriving adolescent animals from play had detrimental effects on social behavior later on (van den Berg et al., 1999; Burleson et al., 2016) and interfered with the neuronal development of the fronto-cortical regions (Bell et al., 2010), but that providing brief daily social contact could partially alleviate effects of isolation rearing (Einon et al., 1978). In particular the reciprocity in a play fight, i.e., the active engagement of the play partner, has been identified as the main rewarding component of these social interactions (Pellis and McKenna, 1995). In our experimental approach, adolescent Wistar animals are deprived of these reciprocal social experiences by being subjected to playful encounters with rats from the inbred Fischer 344 strain. We could recently show that Fischer animals differ profoundly in their quality of social play from outbred strains, such as Wistar or Sprague Dawley. Fischer rats show a diminished responsiveness upon playful attacks, employ different defensive strategies and show increased locomotor play. Hence, during cross-strain playful encounters female adolescent Wistar rats receive only few playful responses from their Fischer partner while increasing their own attack frequency (Schneider et al., 2016). Therefore, Fischer partners do not adequately accommodate the need for social play in Wistar rats, since they negate the most rewarding aspects of social play (Pellis and McKenna, 1995).

In our previously used model, pairing of one female Wistar rat with either one same strain partner or one inadequate Fischer play partner throughout adolescence, induced lasting changes in pain sensitivity and CB1 receptor (CB1R) protein levels, implicating the endocannabinoid (eCB) system as a potential modulator of social rejection experiences (Schneider et al., 2014). However, social skills remained unaffected in adulthood. Based on these previous findings, we refined our initial approach: instead of rearing in pairs, adolescent female Wistar rats were now reared within a group of Fischer animals (inadequate social rearing condition, ISR), or adequately within a group of same-strain partners (control). In adulthood all Wistar rats were then screened for changes in social behavior, pain perception, and alterations in the eCB system in brain regions that are part of the so called social brain (amygdala, striatum and prefrontal cortex; O’Connell and Hofmann, 2011) and were previously found to be affected (Schneider et al., 2014).

## Materials and Methods

### Subjects

**Figure 1:**
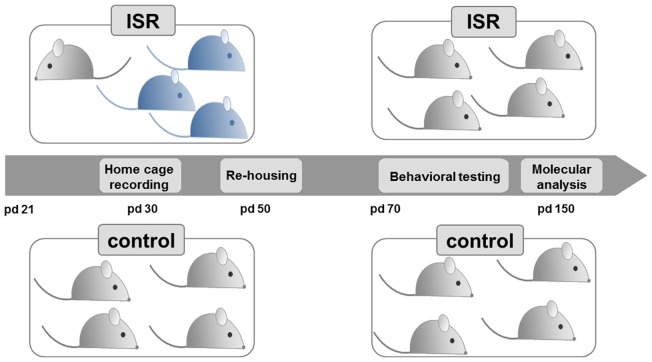
**Experimental design.** Female Wistar rats (light gray) were either reared with same-strain partners during adolescence, from postnatal day (pd) 21–50, considered as adequate social rearing (control), or with three age-matched rats from the Fischer strain (blue), considered as inadequate social rearing (ISR). Beginning on pd 50, all Wistar rats were again group housed with same strain partners and only the Wistar rats of both social rearing conditions (control and ISR) were further examined. Behavioral testing (pd 70–150) and molecular analysis (pd 150) were performed in adult animals.

Female (*n* = 16) and male (*n* = 8) Wistar RccHan rats were purchased from Harlan Laboratories (Venray, Netherlands) and bred together (two females with one male) 1 week after arrival at our facility. Pups were weaned on postnatal day (pd) 21 and only females (*n* = 89, from 162 pups total) were randomly assigned to the experimental groups according to the study design (Figure 1). Age-matched Fischer 344 rearing partners (*n* = 129) for the inadequate rearing groups were purchased (Charles River, Sulzfeld, Germany) at pd 21.

Littermates were not reared together if possible, or were matched across the rearing conditions (<20% littermates per rearing condition). Animals were housed in groups of 4–5 under standard conditions (Makrolon Type IV cages) on a 12 h light-dark cycle (lights on 07:00–19:00) with free access to water and standard lab chow. All experiments were approved by the local animal care committee (Regierungspräsidium Karlsruhe, Referat 35, Karlsruhe, Germany) following the guidelines of the European Union (2010/63/EU).

#### Inadequate and Adequate Social Rearing (Peer-Rejection Paradigm)

The study design (Figure 1) was based on our previous model (Schneider et al., 2014), where we examined long-term consequences of inadequate rearing in pairs. For the present study, female Wistar rats were subjected to the different rearing conditions in groups throughout adolescence directly after weaning (pd 21–50). Control condition: rats were reared within a group of four or five female Wistar rats (preliminary testing in our lab indicated no behavioral differences between group housed animals of four or five); ISR condition: one Wistar animal was reared with three age-matched, female Fischer rats (total group size of four). Three cohorts of animals were used for the present study: cohort 1 for behavioral analysis (control: *n* = 18; ISR: *n* = 15; except for object recognition: only control/ISR: *n* = 10 of cohort 1 were used), cohort 2 for molecular analysis (control/ISR: *n* = 20) and cohort 3 for repeated measurement of corticosterone levels during adolescence and in adulthood (pd 100; control/ISR: *n* = 8).

From pd 50 on, all Wistar rats (controls and ISR animals) were re-grouped with unfamiliar female Wistar rats from the same condition, thereby terminating the ISR condition. Subsequently, the behavioral assessment (cohort 1) was performed in adult rats beginning from pd 70 on (until pd 150), while females of cohort 2 were left undisturbed until day 150 for molecular analysis.

### Social Play Behavior

Home cage behavior was videotaped on pd 30 and 35 to analyze social play in a number of randomly selected animals from both rearing conditions (control: *n* = 9; ISR: *n* = 8) as described previously (Schneider et al., 2014). Both days were chosen since high occurrences of social play have been reported around this age (Pellis et al., 1997) and to enable a direct comparison with our previous study (Schneider et al., 2014) as well as monitor for possible behavioral adaptations.

Animals were videotaped on pd 30 and 35 in their home cage for 1 h at the beginning of the dark phase (under red light). Social play behavior as well as locomotor play was subsequently quantified offline by an experienced experimenter blind to the condition. In order to identify similar periods of intense play for all test groups, the total time recorded (60 min) was pre-screened for the occurrence of play behavior and only one 15 min time span with a peak in playful activities was chosen for further detailed frame-by-frame analysis for each particular group. The following behavioral elements were quantified by a trained observer (for detailed description, see also Schneider and Koch, 2005; Schneider et al., 2014, 2016): (A) Social play behavior: (1) Pinning: one animal is lying on its back with the partner standing over him. Since both animals are involved actively in this behavior pinning was counted for both of them; (2) Attacks: one animal brings the tip of his snout either in contact with or close to the nape of its play partner. Attacks were scored separately when initiated and when received; and (3) Response: including the withdrawal of the nape area by a rat upon attack; the so called complete rotation (upon contact the recipient rotates around its longitudinal axis, cephalocaudally, to a supine position); so called partial rotation (upon contact the recipient rotates around its longitudinal axis, and maintains a standing position on its hind feet) and evasion (the recipient runs, leaps or swerves away from the attacker). Beside the frequency of social play behaviors, the percentage of receiving a response upon attacks/responding upon attacks (%) was analyzed; i.e., receiving an appropriate playful response/giving an appropriate playful response to an attack (i.e., defensive reactions, including evasion, or initiation of a counter attack) to the total number of initiated/received attacks was calculated respectively for each test animal. (B) Locomotor play: locomotor play involves locomotor-rotational movements (i.e., undirected running, galloping and jumps and jumping to and climbing on the grid of the cage lid) which are also used in antipredator behavior and occur as sporadic actions (Fagen, 1981; Pellis and Pellis, 1983).

### Behavioral Testing

Behavioral testing took place between 10:00–16:00 with at least 2 days break between tests, and was videotaped and evaluated offline by a trained experimenter blind to group assignment. The estrous cycle of all post-pubertal animals was monitored, but not pharmacologically synchronized.

### Open Field, Elevated Plus Maze (EPM), Light/Dark Emergence Test (EMT) and Object Recognition Memory

Behavioral testing in these tasks was performed as previously described (Goepfrich et al., 2013).

#### Open Field

Locomotor activity was measured in an open field apparatus, which consisted of four equal arenas (51 cm × 51 cm × 50 cm), made of dark gray PVC. The set up allowed for the parallel measurement of the distance traveled [cm], which was digitally recorded and analyzed with the behavioral observation program Viewer^2^ (Biobserve GmbH, Bonn, Germany). The test was started by placing the animals in the center of the arena and ran for 30 min with a light intensity of 50 lx.

#### Elevated Plus Maze (EPM)

The EPM is a plus-shaped apparatus consisting of dark gray PVC with two open arms (12 cm × 50 cm) and two enclosed arms (12 cm × 50 cm × 50 cm) surrounding a middle platform (12 cm × 12 cm) 50 cm above the floor. At the beginning of each trial, the rat was gently placed on the middle platform facing a closed arm and allowed to explore the maze for 5 min (90 lx). The following behaviors were scored in the subsequent video analysis: time spent in open and closed arms [s], number of entries into open or closed arms (an entry was defined as all four paws in a particular arm), head dips and risk assessment (only head or forepaws are placed in an open arm without concomitant movement of the hind legs, even if the rat subsequently entered the arm) and the percentage of time spent in open arms (open arm time/(open + closed arm time) × 100) was calculated as well.

#### Light/Dark Emergence Test (EMT)

The EMT apparatus consisted of two compartments separated by a dividing wall with a 10 cm × 15 cm wide opening enabling free movement between the smaller, dark compartment (i.e., the start box) with black walls (25 cm × 25 cm × 40 cm) which could be closed by a lid, and the larger, brightly illuminated (90 lx) compartment with gray walls (25 cm × 50 cm × 40 cm). Rats were initially placed in the closed dark compartment and after 1 min given access to the whole area to freely explore for 5 min. The following video analysis scored the latency of the animals to emerge into the lit compartment [s] (an entry was defined as all four paws in compartment), the emergence frequency, the duration of time spent in the lit compartment [s], the number of rearings, and risk assessment behavior (only head or forepaws are placed in the open compartment without concomitant movement of the hind legs, even if the rat subsequently entered the area).

#### Object Recognition Memory

The test consisted of an initial 3 min sample phase (P1) and a 3 min discrimination phase (P2) which were separated by an intertrial interval (ITI) of 15 min. The objects to be discriminated were made of metal, ceramic, or glass and existed in multiple copies. All objects and the test arena were cleaned with 70% ethanol and thoroughly dried before and during testing. Preliminary testing in our lab indicated an equal attractiveness of all objects chosen for this test to the animals. During P1, the rat was placed in the center of the open field and exposed to two identical unknown objects (A). Afterwards, the rat was returned to the home cage and the objects were cleaned and dried. In P2 the rat was returned and now exposed to the familiar object A′ (an identical copy of the object presented in P1) and a novel test object (B). Exploration of the objects (sniffing, touching with whiskers, and licking) was recorded during P1 and P2. The discrimination between the exploration time of the novel object vs. the familiar one was expressed as percentage of the total exploration time of both conspecifics during P2 [100/(A′+B) × B] while for the discrimination index the exploration times in P2 were subtracted (B − A′).

### Social Interaction and Recognition Memory

Social recognition and interaction with an unfamiliar juvenile female social partner were assessed in an open field as previously described (Schneider et al., 2008). Social interaction with an unfamiliar social partner was assessed for 5 min in the open field. Test animals as well as the juvenile female social partners (6–7 weeks of age) were habituated to the test arena for 15 min, 24 h before testing. The frequency of the following behaviors (see description below) was quantified for the test rat only: (I) Social behavior; (1) Contact behavior: (a) social grooming (chewing or licking of the partner’s fur), (b) crawling over/under the partner’s body. (2) Social exploration: (a) anogenital and (b) non-anogenital investigation of the partner (sniffing or licking the anogenital or any part except for the anogenital area, respectively) and (c) approaching and/or following the social partner throughout the test arena. (II) Social evasion: active avoidance of social contact initiated by the social partner. (III) Self-grooming: sequence of licking the forepaws and rubbing them over the head or licking/biting its own fur.

The initial 5 min social interaction period with the unfamiliar social partner (A) served as sample phase (P1) for the social recognition test 2. In the subsequent test for social recognition memory a second unfamiliar juvenile (B) was introduced into the test (P2). After the 15 min ITI the familiar (A′) and a novel social partner (B) were presented to the experimental animal for 3 min in P2. The time for social investigation (anogenital exploration, non-anogenital exploration and approach/following) were recorded during P1 and P2. For the calculation of social discrimination percentage the exploration time of the novel conspecific was expressed as percentage of the total exploration time of both conspecifics during P2 [100/(A′+B) × B], while for the discrimination index the exploration times in P2 were subtracted (B − A′).

### Social Transmission of Food Preference

In this test adapted from Galef (2003), one rat per cage (demonstrator) was given access to a flavored food (cinnamon/vanilla scented shortbread crumbs). Demonstrator and observer animals were habituated to the experimental room and the single cages (Macrolon^TM^ Typ III) on 2 days prior to the experiment. Pre-tests in our lab showed that both scents were equally preferred and to ensure objective rating the scents were counterbalanced in the test. During the 12 h period prior to the test all animals were food restricted to 12 g per rat to enhance motivation for food consumption. The demonstrator was presented with 10 g novel scented crumbs and typically consumed 1.5–2 g in 15 min. The demonstrator was allowed to rejoin its cage mates (observers) to communicate this new food source by the odor on its breath/whiskers, prompting a preference in the observers. Following a 3 h interaction interval, the observer animals were transferred to single cages and given the choice between the socially transmitted (e.g., cinnamon) and unfamiliar (e.g., vanilla) scented food (scents were counterbalanced). Observer rats were presented with 5 g food per flavor for 5 min, consuming 1 g on average, the amount of shortbread crumbs consumed during testing was measured and the preference for the transferred food was calculated in percentage of the total amount of food consumed.

### Hot Plate Test

Pain sensitivity was measured at a constant temperature of 52.5 ± 0.1°C on a hot plate apparatus (Ugo Basil, Varese, Italy) as previously described (Schneider et al., 2014). Behavior was videotaped and analyzed for the latency [s] of the first heat-provoked response defined as either a foot shake or stamping, paw licking or jumping off of the platform.

### Endocrine Analysis

#### Blood Sampling and Corticosterone Measurement

During adolescence blood samples for the analysis of stress hormone levels were taken from cohort 3 every 6 days at the same time point (4 h prior to light off), starting on pd 22. For sampling on pd 100, animals were prescreened for estrous stage (at the beginning of the light period) so that only females in the diestrus stage were sampled. Samples (30 μl) were taken by puncture of the saphenous vein, in a time period of 3 min for each animal and in a randomized order. Samples were prepared as per the manufacturers’ protocol (125I RIA double antibody corticosterone Kit, MP Biomedicals LLC, Orangeburg, SC, USA).

#### Molecular Analysis

Rats were briefly anesthetized with CO_2_ and swiftly decapitated. Brains were removed and either immediately shock-frozen in 2-methylbutan for later [^35^S]GTPγS binding (control/ISR *n* = 10) or quickly dissected (i.e., mPFC, striatum, thalamus and amygdala; control/ISR *n* = 10): samples from the right hemisphere were homogenized for Western Blot analysis, while samples from the left hemisphere were frozen in liquid nitrogen for analysis of anandamide (AEA) and 2-arachidonoylglycerol (2-AG). Coronal brain slices were cut as serials on a cryostat (12 μm), thaw-mounted on glass slides. All samples were stored at −80°C before processing.

#### Western Blot

Primary antibodies used were: fatty acid amide hydrolase (FAAH) and monoacylglycerol lipase (MAGL; cat.no.101600/100035, Cayman, Ann Arbor, MI, USA) and CB1R (IMG-pAb001, Immunogenic, Zug, Switzerland). The fluorescent secondary antibodies that were used made simultaneous detection of MAGL/FAAH/CB1R and ß-Actin possible due to reacting to two different fluorescent wavelengths (donkey-anti-rabbit 800, cat.no.926-32213 and donkey-anti-goat 680, cat.no.926-68024 LiCor Biosciences, Bad Homburg, Germany). Band density was quantified with the Odyssey Imaging System and Image Studio software (LiCor Biosciences). Background-corrected values of FAAH (single band, 63 kDa), MAGL (doublet (Long et al., 2009), 33 and 35 kDa) and CB1R (single band, 53 kDa) were corrected for the corresponding ß-Actin contents and expressed as percentage changes to control group.

Protein content was measured by Bradford Protein Assay (BioRad Laboratories GmbH, Munich, Germany) and protein samples (25 μg) were then separated by electrophoresis at 200 V in NuPage^®^ Bis-Tris 4–12% gels (Invitrogen, Darmstadt, Germany). Separated proteins were then blotted (400 mA for 90 min) onto PVDF membranes (BioRad Laboratories, Munich, Germany) and blocked with either Odyssey^TM^ blocking buffer/TBS solution (LiCor Biosciences GmbH, Germany) at room temperature (RT) for 30 min (FAAH) or 1 h (MAGL) or in 2.5% nonfat dry milk/TBS solution at RT for 1 h (CB1R).

Membranes were then incubated with primary antibodies against either FAAH (1:1000) or with rabbit polyclonal anti-MAGL antibody (1:500) at 4°C overnight (Odyssey^TM^ blocking buffer/TBS solution) or against CB1R (rabbit polyclonal, 1:1000) for 24 h at 4°C (2.5% nonfat dry milk/TBS solution). The loading control protein was ß-Actin (goat polyclonal, 1:2000, sc-1615, Santa Cruz Biotechnology Inc., Heidelberg, Germany). The secondary antibodies were incubated in Odyssey^TM^ blocking buffer/TBS solution for 1 h at RT. Secondary antibodies were used as follows: donkey-anti-rabbit 800 for FAAH and CB1R, respectively 1:10,000, and MAGL 1:15,000, and donkey-anti-goat 680 for ß-Actin (1:10,000).

#### Agonist-Stimulated [^35^S]GTPγS Binding

The protocol was used as detailed by Rojo et al. (2011). Briefly, after defrosting (30 min at RT) slides were prepared in pre-incubated buffer (containing 50 mM Tris-HCl, 3 mM MgCl_2_, 0.2 mM EGTA, 100 mM NaCl, 2 mM GDP, 1 mM DTT, 0.5% free fatty acid (ffa) BSA, pH 7.7) for 30 min at RT, followed by an incubation for 2 h at 27°C in a second batch pre-incubation buffer with added 10 μM/ml deaminase (Sigma Aldrich, Taufkirchen, Germany) and 0.04 nM [35S]GTPγS (Perkin Elmer, Rodgau, Germany). Three conditions were assessed: basal (buffer only), non-specific binding [+10 μM unlabeled GTPγS (Sigma Aldrich)], and CB1R agonist stimulated [+10 μM CP 55, 940 (LGC Standards, Wesel, Germany)]. Incubation was stopped in ice-cold wash buffer (50 mM Tris HCl buffer, pH 7.4 containing 0.5% ffa BSA) followed by dipping in cold ultrapure water. Subsequently slides were dried (overnight at 4°C) before being placed in photo cassettes with [14C] standards (American Radiolabeled Chemicals, Saint Louis, MO, USA) with Kodak BioMax MR film (Kodak, Rochester, NY, USA), exposed for 48 h at 4°C. Films were developed, digitized and later analyzed with ImageJ software (Schneider et al., 2012). Gray values were analyzed (calibrated to [14C] standards) and regions corresponding to Western Blot and eCB analysis were measured (3 sections per region) for each animal. Non-specific activity was subtracted from basal and CP-induced activity measures to gain specific activity. Percentage increase of CP-induced activity over basal activity (% stimulation over basal) was calculated as follows: % stimulation over basal activity = [CP (specific) − basal (specific)]/basal (specific)*100.

#### eCB Analysis

For eCBs extraction brain tissues were first transferred to extraction tubes containing cold steel beads. Spiking solution of deuterated eCBs in acetonitrile was mixed with 0.1 M formic acid, which served as homogenization buffer, and rapidly pipetted to the extraction tubes using an automated pipetting procedure (ThermoScientific; Lomazzo et al., 2015). Ethylacetate/hexane (9:1) for eCBs extraction was then manually pipetted. Tissues were homogenized for 30 s or 1 min (the homogenization time was adapted to the tissue type,) at 30 Hz with a tissue lyser (Qiagen). Homogenates were then centrifuged at 5000 g, 4°C for 15 min and then kept for 30 min at −20°C to freeze the aqueous phase. The upper organic phase was recovered in microtitter plates, evaporated to dryness, and the extracts reconstituted in 50 μl water:acetonitrile (1:1) using the automated pippetter. Throughout the extraction procedure, the tubes, plates, beads, etc., were invariably precooled and kept at 4°C. The samples were as well, invariably kept on ice throughout the entire extraction procedure to prevent artificial alterations of endogeneous eCB levels originating from enzymatic or chemical degradation and/or *ex vivo* synthesis of eCBs. The amounts of internal standards and concentration range of calibration curves were tailored to each investigated brain region. eCBs were qualitatively and quantitatively determined using Liquid Chromatography (LC)/Multiple Reaction Monitoring (MRM). The LC and MRM conditions were as previously described (Wenzel et al., 2014; Lomazzo et al., 2015). The water phase resulting from the liquid-liquid extraction of lipids was used for protein determination using a BCA assay. The CBs amounts determined by LC/MS were normalized to the tissue weight.

#### Statistical Analysis

Differences between the two rearing conditions were analyzed by Student’s *t*-tests, and in addition one-sample *t*-tests were performed to analyze the preferences against chance level (50%). The repeated measure of plasma corticosterone levels was analyzed by repeated measure analysis of variance [RM analysis of variance (ANOVA)].

All data were calculated using SPSS22 (IBM Deutschland GmbH, Ehningen) and are expressed as mean ± SEM. The level of statistical significance was defined as *p* < 0.05.

## Results

### Adolescent Social Play Behavior

**Figure 2:**
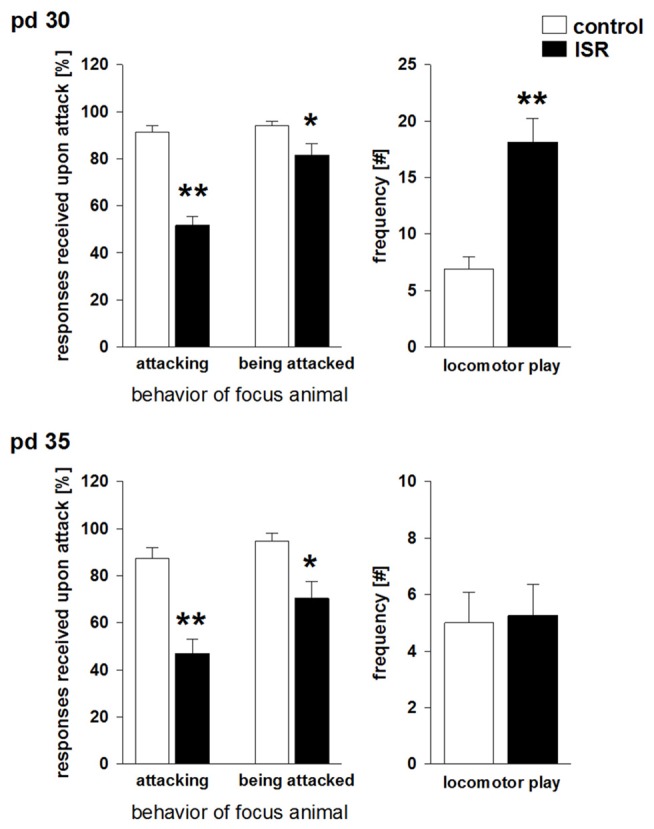
**Social play behavior during adolescence on pd 30 and pd 35 in Wistar females subjected to adequate (control) or ISR conditions.** The probability of a defensive response received by the play partner after an attack by the focus animal was significantly reduced in females of the ISR condition on both time points. In addition, ISR Wistar rats also responded significantly less when being attacked by the play partner than controls which was further reduced on pd 35. Locomotor play was significantly increased in ISR animals compared to control rats only on pd 30. Data are indicated as means ± SEM (control: *n* = 9; ISR: *n* = 8; **P ≤ 0.001; *P ≤ 0.05).

When analyzed on pd 30 (Figure 2), ISR rats received a significantly lower number of appropriate play responses upon playfully attacking a cage mate (e.g., Fischer rat) than did Wistar rats in the control condition (*T*_15_ = 8.474; *P* < 0.001). In turn, ISR females themselves did respond significantly less when cage mates (e.g., Fischer rat) directed playful attacks at them than was found for the Wistar rats in the control condition (*T*_15_ = 2.481; *P* = 0.025).

Apart from this altered responsiveness upon play solicitation a significant increase in locomotor play was found in ISR rats compared to controls (*T*_15_ = −4.873; *P* < 0.001). Furthermore, the number of attacks initiated by Wistar females was significantly elevated (*T*_15_ = −7.474; *P* < 0.001) in the ISR condition (Table 1). A trend for a decrease in the frequency of attacks received by the play partners was found in ISR rats compared to controls (*T*_15_ = 1.802; *P* = 0.092). The number of pinnings did not differ between the groups (*T*_15_ = 0.554; *P* = 0.588; Table 1). On pd 35 (Figure 2) animals in the control condition again showed a high responsiveness to playful attacks while ISR rats received significantly less adequate responses attacking a play partner: *T*_15_ = 5.517; *P* < 0.001; being attacked by a play partner: *T*_15_ = 3.214; *P* = 0.006). However, instances of locomotor play did not differ in frequency between both conditions (*T*_15_ = −0.161; *P* = 0.874). Females in the ISR condition showed a trend for an increased frequency of attacks toward their cage mates (*T*_15_ = −1.921; *P* = 0.074) while the number of received attacks (*T*_15_ = 0.866; *P* = 0.400) and pinnings (*T*_15_ = −0.639; *P* = 0.533) did not differ compared to controls (Table 1).

**Table 1:** **Social play behavior in adolescent (on postnatal day (pd) 30 and pd 35) control (adequate social rearing) and inadequate social rearing (ISR) animals**.

Play frequency	Control	ISR
pd 30 attacks initiated	15.77 ± 1.26	31.03 ± 1.64**
pd 30 attacks received	16.00 ± 1.17	13.00 ± 1.18^#^
pd 30 pinning	17.67 ± 0.91	17.00 ± 0.76
pd 35 attacks initiated	10.56 ± 0.9	17.5 ± 3.71^#^
pd 35 attacks received	9.33 ± 0.97	8.25 ± 0.75
pd 35 pinning	14.11 ± 2.01	16.25 ± 2.74

Data are indicated as means ± SEM (control: n = 9; ISR: n = 8; **P < 0.001; ^#^P ≤ 0.1).

### Corticosterone Measurement during Adolescence

**Figure 3:**
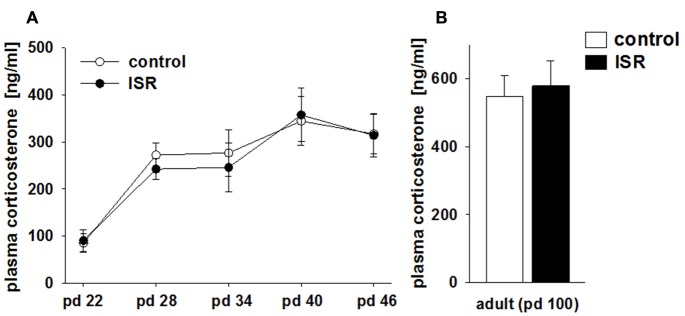
**Comparison of blood corticosterone levels in adolescent (A) and adult (B) females of the adequate (control) and the ISR condition.** Baseline corticosterone levels in rats of both rearing conditions did not differ over the course of adolescence between the two rearing conditions. Data are indicated as means ± SEM (control/ISR: *n* = 8 each).

Baseline blood corticosterone levels in rats of both rearing conditions did not differ over the course of adolescence at any time point tested, although corticosterone levels did rise significantly in both groups over time (Figure 3A; rearing condition: *F*_(1,86)_ = 0.42; *P* = 0.619; age: *F*_(4,86)_ = 9.57, *P* < 0.001; rearing condition × age: *F*_(4,86)_ = 0.16, *P* = 0.958). Furthermore, no group differences in corticosterone levels were found in adulthood (pd 100; Figure 3B; *P* = 0.958).

### Behavioral Assessments in Adulthood

No significant group differences were detected between ISR and control rats for locomotor activity in an open field (distance traveled: control = 5150.41 ± 272.85 cm; ISR = 5248.30 ± 273.77 cm; *P* > 0.1) and performance in the EMT test (Table 2; *P* > 0.1). Most variables of EPM testing also did not differ between the groups (Table 2; *P* > 0.1), except for the higher number of closed arm entries in ISR rats (*T*_31_ = −2.243; *P* = 0.032).

**Table 2:** **Behavioral performance of adult control (adequate social rearing) and ISR animals on the elevated plus-maze (EPM) and the light/dark emergence test (EMT)**.

		Control	ISR
**EPM**	Open arm time [%]	14.39 ± 2.63	17.03 ± 3.38
	Open arm entries [%]	19.87 ± 3.03	19.01 ± 2.86
	Closed arm entries	7.83 ± 0.66	10.07 ± 0.75*
	Head dips	9.78 ± 1.30	9.67 ± 1.21
	Risk assessment	7.06 ± 0.72	8.67 ± 0.67
**EMT**	Emergence latency [s]	89.88 ± 20.83	97.93 ± 23.86
	Entries	4.11 ± 0.51	4.60 ± 0.67
	Time spent [s]	77.72 ± 11.68	73.4 ± 12.65
	Risk Assessment	6.11 ± 0.46	6.20 ± 0.90

Data are indicated as means ± SEM (control: n = 18; ISR: n = 15; *P ≤ 0.05).

**Figure 4:**
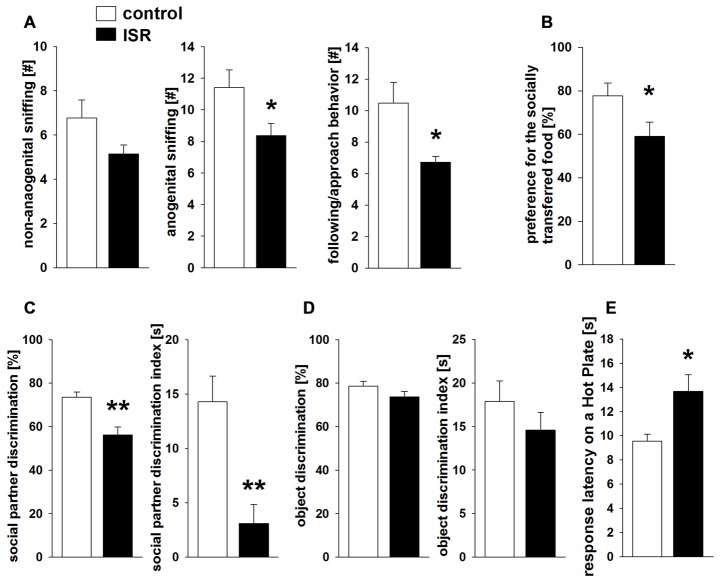
**Behavioral performance in adult rats after adequate (control) or ISR experiences in adolescence.** During social interaction testing **(A)** anogenital sniffing and following/approach behavior were significantly reduced in ISR rats. Similarly, rats of the ISR condition showed a deficient performance in the social food transfer task and showed a decreased preference for the socially transmitted food source. **(B)** The ability to recognize social partners was significantly affected in ISR rats, as indicated by a decrease in % discrimination and the discrimination index compared to controls. **(C)** Object recognition abilities (% discrimination or discrimination index) did not differ between females of either rearing condition. **(D)** Pain sensitivity to a thermal stimulus measured in adult rats of the control and the ISR condition **(E)** showed higher response latency on the hot plate in ISR rats, indicating significantly decreased pain sensitivity. Data are indicated as means ± SEM (control: *n* = 18; ISR: *n* = 15; object recognition: control/ISR *n* = 10; **P ≤ 0.001; *P ≤ 0.05).

In the social interaction test (Figure 4A), ISR rats showed significantly reduced anogenital sniffing (*T*_31_ = 2.244; *P* = 0.032) and following/approach behavior (*T*_31_ = 2.634; *P* = 0.013) compared to controls, while non-anogenital sniffing did not differ (*T*_31_ = 1.568; *P* > 0.1). No differences were observed in contact behavior (*P* > 0.1; values: controls = 0.41 ± 0.15; ISR = 0.93 ± 0.34), evasion (*P* > 0.1; values: controls = 1.12 ± 0.32; ISR = 0.71 ± 0.38) or self-grooming (*P* > 0.1; values: controls = 0.41 ± 0.12; ISR = 0.14 ± 0.10). ISR animals displayed significantly less preference for the socially transferred food (Figure 4B; *T*_24_ = 2.125; *P* = 0.044) compared to controls. Social recognition (Figure 4C) was also significantly reduced in ISR rats (percentage discrimination: *T*_31_ = 4.064; *P* < 0.001; discrimination index: *T*_31_ = 3.678; *P* < 0.001) although, initial exploration duration during P1 did not differ (*T*_18_ = −0.057; *P* = 0.955; values: controls = 29.35 ± 3.76 s; ISR = 29.57 ± 3.03 s). No group differences were detected in object recognition testing (Figure 4D; percentage discrimination: *T*_18_ = 1.668; *P* = 0.153; discrimination index: *T*_18_ = 1.058; *P* = 0.305; P1 exploration: *P* > 0.1; values: controls = 26.89 ± 1.87 s; ISR = 22.50 ± 1.84 s).

Finally, the measurement of thermal pain sensitivity on a hot plate (Figure 4E) revealed a significantly longer response-latency in ISR rats compared to controls (*T*_31_ = −2.906; *P* = 0.007).

### Molecular Analysis

**Figure 5:**
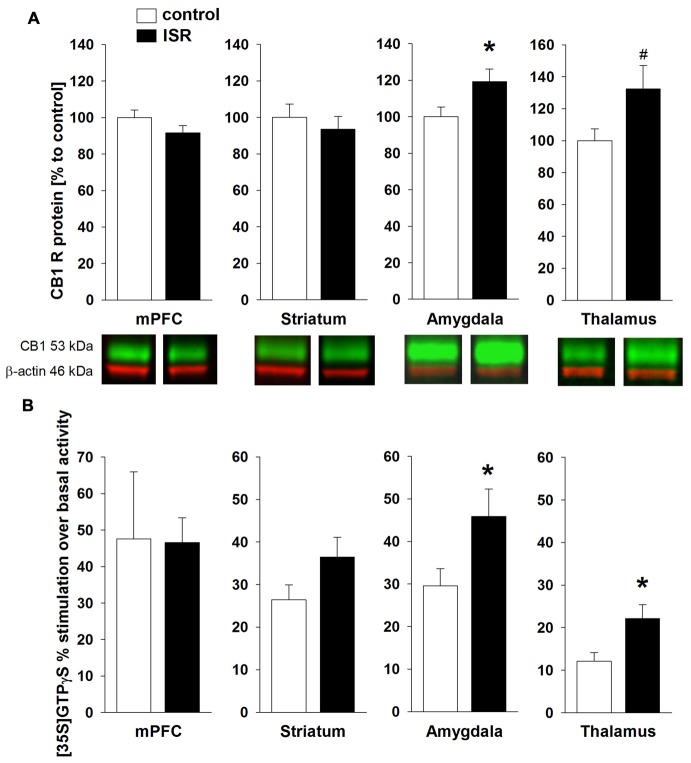
**CB1 receptor (CB1R) protein levels in adult rats from the adequate (control) and the ISR condition.** CB1R protein levels **(A)** were increased in the amygdala of ISR rats. A trend (*p* = 0.06) for increased CB1R levels was detected in the thalamus of ISR rats, with no further changes in the medial prefrontal cortex (mPFC) and striatum. CP 55, 940 stimulated (% over basal) [^35^S]GTPγS binding **(B)** was significantly in the amygdala and thalamus (control *n* = 10; ISR *n* = 9) of the ISR females, while remaining unchanged in mPFC (control *n* = 8; ISR *n* = 9) and striatum. Data are indicated as means ± SEM (control/ISR: *n* = 10 each; *P ≤ 0.05; ^#^*P* ≤ 0.1).

CB1R protein levels (Figure 5A) were significantly higher in the amygdala of ISR compared to control rats (*T*_18_ = −2.204; *P* = 0.041), a trend for such an increase was detected in the thalamus (*T*_18_ = −1.975; *P* = 0.064). No differences between both rearing conditions were detected in the mPFC (*T*_18_ = 1.480; *P* = 0.156) or striatum (*T*_18_ = 0.644; *P* = 0.528). Interestingly, a similar pattern was found for the agonist-stimulated [^35^S]GTPγS binding (Figure 5B) with significantly higher CB1R activity over basal stimulation in the amygdala (*T*_18_ = −2.155; *P* = 0.045) and thalamus (*T*_18_ = −2.626; *P* = 0.018) in ISR than in control females, whereas no differences were found in the mPFC (*T*_18_ = 0.051; *P* = 0.960) and striatum (*T*_18_ = −1.723; *P* = 0.102).

**Figure 6:**
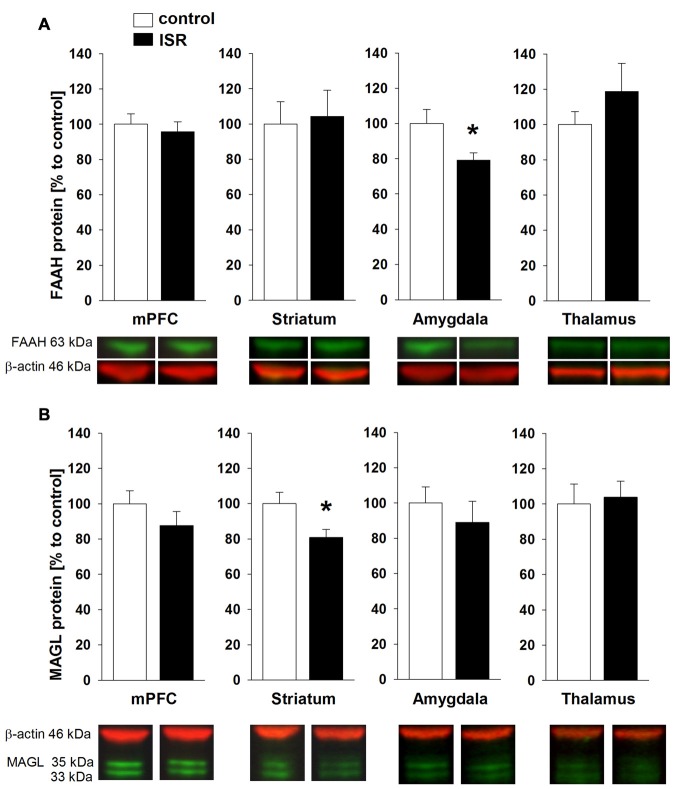
**Fatty acid amide hydrolase (FAAH) and monoacylglycerol lipase (MAGL) protein levels in adult rats from the adequate (control) and the ISR condition.** FAAH protein levels **(A)** were decreased specifically in the amygdala of ISR rats but were unchanged in the mPFC, striatum and thalamus. MAGL protein levels **(B)** were significantly reduced in the striatum of ISR rats compared to control rats but were unchanged in the mPFC, amygdala and thalamus. Data are indicated as means ± SEM (control/ISR: *n* = 10 each; *P ≤ 0.05).

A significant reduction of FAAH was detected in the amygdala of ISR rats compared to controls (Figure 6A; *T*_18_ = 2.334; *P* = 0.031). FAAH levels did not differ in any of the further analyzed regions between control and ISR animals (mPFC: *T*_18_ = 0.520; *P* = 0.610; *T*_18_ = −0.226; striatum: *P* = 0.824; *T*_18_ = −1.058; thalamus: *P* = 0.304). However, striatal levels of MAGL (Figure 6B) were significantly reduced in females from the ISR condition (*T*_18_ = 2.452; *P* = 0.025), while levels in the mPFC (*T*_18_ = 1.133; *P* = 0.272), amygdala (*T*_18_ = 0.726; *P* = 0.477) and thalamus (*T*_18_ = −0.1621; *P* = 0.797) remained unchanged to controls.

**Figure 7:**
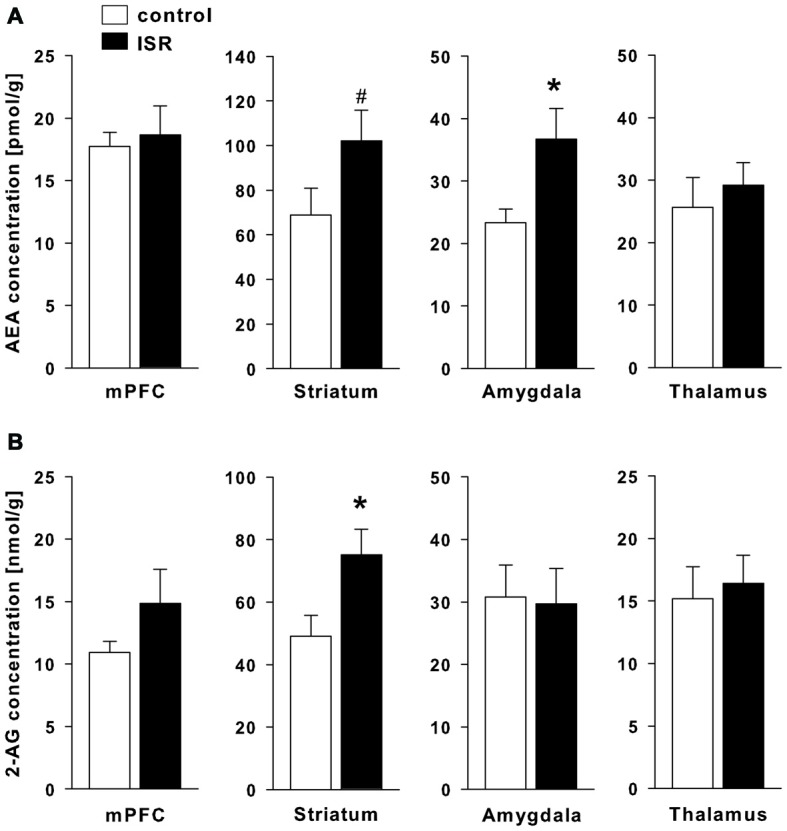
**Endocannabinoid (eCB) levels in adult rats from the adequate (control) and the ISR condition.** Levels of the eCB analysis of anandamide (AEA; **A)** were significantly increased in the amygdala and the striatum, while levels of 2-AG **(B)** were increased in the striatum of ISR animals as compared to controls. No further significant changes were observed for other brain regions tested (mPFC and thalamus). Data are indicated as means ± SEM (control/ISR: *n* = 10 each; *P ≤ 0.05, ^#^*P* ≤ 0.1).

A significant elevation of the eCB AEA (Figure 7A) was found in the amygdala of ISR rats (*T*_18_ = −2.480; *P* = 0.023) and a trend for higher AEA concentrations was found in the striatum (*T*_18_ = −1.812; *P* = 0.087). AEA concentrations did not differ in the mPFC (*T*_18_ = −0.155; *P* = 0.727) nor the thalamus (*T*_18_ = −0.599; *P* = 0.557). The levels of the second major eCB 2-AG (Figure 7B) were significantly increased in the striatum (*T*_18_ = −2.477; *P* = 0.023) of ISR females, while no group differences were found for other regions tested (mPFC: *T*_18_ = −1.366; *P* = 0.189; amygdala: *T*_18_ = 0.145; *P* = 0.887; thalamus: *T*_18_ = −0.355; *P* = 0.727).

## Discussion

In the present study, we refined our initial approach to model long-term peer-rejection in adolescent female Wistar rats. The present results demonstrate that the inability to adequately engage in playful peer-interaction during adolescence results in lasting impairments in pain sensitivity and a variety of social abilities in adulthood by decreasing social interaction behavior, social memory and the processing of socially transmitted information. No lasting effects were observed on object recognition memory, locomotor activity and anxiety-related behaviors. Although, increased closed arm entries were observed in ISR rats in the EPM, this effect appeared transient since no increased activity in a novel environment was detected in the open field and the EMT. Molecular analysis revealed a significantly affected central eCB system in adult ISR animals, with changes in the amygdala, striatum, and in the thalamus.

We previously employed a dyadic design to model long-term peer-rejection through inadequate playful interaction during adolescence (Schneider et al., 2014), which in contrast to the presently used group design, did not yield any lasting impairments on social interaction behavior, although minor changes in the eCB system and decreased pain sensitivity were detected. Here, female Wistar animals were reared within adequate/inadequate groups, accommodating the apparent need in female rats for housing in groups vs. housing in pairs (Patterson-Kane et al., 2004). Our previous dyadic design may therefore have already affected social behavior in control animals through the lack of a sufficiently social environment and in turn explain why no differences were observed in social skills between ISR and control rats (Schneider et al., 2014). Allowing for social group living normalizes the social behavior in controls while also increasing the “outsider” position of the lone Wistar female. The analysis of social play behavior, assessed during the rearing condition on pd 30 and 35 revealed distinct differences in the quality of playful interactions between the adolescent Wistar ISR and control females, similar to the behavioral findings observed recently in acute inadequate social encounters (Schneider et al., 2016). In particular, only half of the nape attacks initiated by an ISR Wistar rat resulted in a defensive playful response by the Fischer play partners, whereas in the control condition almost all attacks resulted in a defensive, play appropriate response. This is of interest because it is commonly understood that play solicitation (i.e., nape attacks) alone, in the absence of defense by the recipient, does not serve as a sufficient reinforcer for playful interactions and therefore the responsiveness of the play partner is considered crucial for the rewarding nature of social play (Pellis and McKenna, 1995). No differences were observed for attacks received and pinning indicating that the Fischer rats do initiate playful interactions, while the number of attacks initiated was substantially higher in Wistar rats of the ISR condition on both days. This enhanced attack frequency in ISR Wistar rats appears to be directly linked to the non-responsiveness of their Fischer play partners. In order to achieve a similar number of rewarding social play interactions as in the control condition, ISR Wistar rats initiated twice as many attacks. This specific lack of an appropriate and active response of the Fischer play partners upon play solicitation may constitute a major part of the aversiveness during the rearing in our social rejection model which lasts through the main period of playfulness during adolescence (Pellis and Pellis, 1990). This hypothesis is further supported by the pronounced increase in locomotor play in ISR Wistar rats on pd 30, implicating a shift toward playful activities perceived as more rewarding than social play with the Fischer partners. However, on pd 35 differences in locomotor play were no longer observed indicating that behavioral adaptations take place throughout the rearing experience.

No group differences were observed in plasma corticosterone levels during the rearing condition, indicating that the general stress load did not induce a pronounced physiological stress-response during adolescence. Hence, the persistent and profound behavioral and molecular effects found in females of the ISR condition may not be mediated by increased stress levels due to the inadequate group housing over adolescence.

Different lasting behavioral alterations of the adolescent rejection experiences were observed in adulthood. During social interaction testing ISR animals showed a decrease in anogenital exploration and approach behavior compared to controls. Both variables can be linked to a more active and motivational aspect of social exploration while non-anogenital exploration, which has been argued to take less effort (Hamilton et al., 2010), did not differ between groups. No further differences were detected for contact behavior, evasion or self-grooming. Decreased social interaction has been reported as a prominent finding in animal models of social deprivation. Social interaction deficits were mainly reported in male rats, while the isolation condition still lasted (Ferdman et al., 2007; Meng et al., 2010), however isolation rearing in females was found to induce either no (Ferdman et al., 2007) or only minor changes (Hermes et al., 2011). In contrast, in the present study female rats from the ISR condition showed a persistent decrease in social interaction and social short-term memory that was measured around 1 month after termination of the inadequate housing conditions. Similar lasting deficits in social short-term memory have been reported in studies of adverse early-life experiences (e.g., maternal separation, pre-weaning ethanol exposure; Kelly and Tran, 1997; Lévy et al., 2003; Lukas et al., 2011). Notably, the deficit in memory processing in ISR rats was specific to the social domain, since object recognition memory was unaffected. Likewise, short isolation periods in adult rats were shown to specifically affect social recognition but not object recognition memory (Shahar-Gold et al., 2013). Rearing with inadequate play partners also strongly affected the ability to process socially transmitted information in a social food transfer task. ISR rats significantly less preferred the socially transmitted food than controls. Although it could be argued that the deficits in social interaction observed in adult ISR rats may underlie the lack of social learning, it has previously been shown that this paradigm does not depend on the familiarity of the donor and that a brief investigation of the snout area is sufficient to promote the preference for the novel food source (Galef et al., 1988). Similar to the acquisition of social recognition memory, the initial processing of the transmitted odor during social food transfer testing in rodents depends on the amygdala, in particular the basolateral region (Wang et al., 2006; Carballo-Márquez et al., 2009). Finally, animals reared within a group of inadequate social play partners showed a persistent decrease in pain sensitivity, similar to the findings observed in our previous dyadic model (Schneider et al., 2014). ISR rats displayed a higher latency for responding to a thermal stimulus compared to controls, indicating that the inability to adequately engage in social play behavior during adolescence induced hypoalgesia in adulthood.

The eCB system is an ubiquitous neuromodulatory system which modulates various processes of neuroplasticity (Katona and Freund, 2012), social behavior (Schneider et al., 2008; Klugmann et al., 2011) and pain processing (Guindon and Hohmann, 2009). Our findings indicate a lasting increase in eCB signaling in ISR animals, mainly within the amygdala, the striatum and thalamus. We observed enhanced CB1R protein levels and receptor/G protein coupling with concomitant increased levels of AEA in the amygdala of ISR animals compared to controls, while protein levels of the AEA degrading enzyme FAAH were decreased. A trend for increased CB1R levels with accompanied increase in receptor coupling was also detected in the thalamus, whereas no group differences for CB1R were observed in the striatum and the mPFC. Two-AG levels were elevated in the striatum, whereas protein levels of the degrading enzyme MAGL were decreased. The described changes were present 100 days after the cessation of adolescent peer-rejection and the results are partially in line with our previous findings from the dyadic model (Schneider et al., 2014), where we detected an up-regulation of thalamic CB1R and increased FAAH levels in the amygdala. The group design employed in the present study therefore exerts a stronger, albeit similar, lasting effect on various components of the eCB system than rearing in inadequate pairs.

The amygdala is part of the social behavior network (O’Connell and Hofmann, 2011) and eCB signaling within the amygdala is well known to modulate social behavior (Trezza et al., 2012; Seillier et al., 2013) and social memory (Segev and Akirav, 2011). Since we observed profound changes in amygdalar eCB signaling in ISR rats, these region-specific alterations may have contributed to the social learning deficits observed in these animals. Although it has been shown that positive social experiences correlate with an acute elevation of amygdalar AEA levels (Trezza et al., 2012), a pharmacological inhibition of FAAH, resulting in an elevation of AEA, was found to attenuate social interaction (Seillier et al., 2013). We report here that adverse peer-experiences induce a lasting increase in amygdalar CB1R/AEA levels and impair social skills. The additionally found increase in agonist stimulated activity of CB1R in the amygdala and thalamus further indicates a profoundly dysregulated eCB system in ISR females. So far, investigations on the impact of aversive early life experiences on CB1R concentrate on isolation rearing and are often inconsistent (Malone et al., 2008; Robinson et al., 2010; Sciolino et al., 2010), although results indicate that receptor density and activity in amygdala and thalamus remain unaffected by isolation rearing while CB1R coupling is downregulated in the striatum (Zamberletti et al., 2012). The results therefore appear to differ from our peer-rejection model where no changes were detected in striatal CB1R expression and binding. However, we observed alterations in striatal eCB content. Although, not primarily involved in the social behavior network, striatal regions have been implicated in social reward processing (O’Connell and Hofmann, 2011). In human adolescents, increased activity in the striatum and thalamus was also observed following acceptance-feedback from peers (Guyer et al., 2012).

Environmental stressors can activate descending pain-inhibitory systems which suppress pain by inhibiting transmission from nociceptors to the central nervous system. This stress-induced analgesia has been shown to be mediated by AEA signaling in the basolateral amygdala (BLA; Connell et al., 2006; Rea et al., 2013), where CB1R and FAAH immunoreactivity are dense (Gulyas et al., 2004). Increased availability of AEA in the BLA has been suggested to suppress pain perception by activation of CB1R on GABAergic interneurons which would disinhibit output neurons and thereby lead to activation of the descending inhibitory-pain pathway (Connell et al., 2006; Rea et al., 2013). The persistent increase in amygdalar AEA levels in our ISR rats may therefore underlie the decrease in thermal pain sensitivity observed in these animals. Additionally, activation of thalamic CB1R has been shown to contribute to the antinociceptive efficacy of cannabinoids (Martin et al., 1996), which is in line with our findings on enhanced thalamic CB1R levels and activity in ISR animals. Experimentally induced social rejection has also been shown to acutely alter nociception in humans (Borsook and MacDonald, 2010; DeWall et al., 2010; Bernstein and Claypool, 2012). Additionally, altered pain perception has been reported in neuropsychiatric disorders that are linked to early experiences of social rejection, such as BPD (Schmahl et al., 2006) and post-traumatic stress disorder (PTSD; Strigo et al., 2010) and appear to be associated with dysregulated amygdala activity (Kraus et al., 2009).

Recent studies in mice indicated that reducing levels of 2-AG by genetic inactivation of the main 2-AG synthesizing enzyme DAGLα lead to a phenotype of increased anxiety and depressive-like behavior. Furthermore, AEA levels were reduced in a region specific manner, indicating possible crosstalk through a most likely indirect mechanism (Jenniches et al., 2016). Interestingly, social behavior and pain sensitivity (hot plate) in these knock-out mice appeared unaffected. Opposite patterns in region specific (cortical vs. sub-cortical) up- and down-regulation of AEA and 2-AG, respectively, but not necessarily FAAH and MAGL protein levels, were observed in repeated homotypic restrained stress, interpreted as an adaptive mechanism to counteract repeated stress responses (reviewed in Patel and Hillard, 2008). These changes in eCB levels did not affect CB1R density or affinity. In the present study changes were region specific but not complementary, i.e., decrease of 2-AG did not result in increase of AEA in the same region, and anxiety behavior was unaffected. Furthermore, a study comparing the sociability enhancing properties of acetaminophen in three mouse strains found similar CB1R densities but increased stimulated binding in a GTPγS assay in the frontal cortex of strains known to be deficient in social behavior (Gould et al., 2012). Hence, further studies are needed to clarify whether the presently found pattern in complementary up and downregulated ligands and catabolic enzymes are functionally independent as they appear to be brain region specific adaptations of the eCB system, or whether they result from regional crosstalk and interactions with other neurotransmitter systems involved in social motivation and social reward behavior such as the oxytocin or opioid system (Gordon et al., 2011; McCall and Singer, 2012; Smith et al., 2015).

Altered eCB signaling in the amygdala (and to a lesser extent in the striatum and thalamus) may have occurred during the inadequate peer-experience in adolescent rats in order to counteract with this socially aversive environment and subsequently lead to persistent adaptational changes within the eCB system into adulthood (Schneider et al., 2014). Furthermore, we could show recently that pre-treatment with the CB1 antagonist/inverse agonist SR141716 prevented changes in pain sensitivity during acute social rejection encounters, thereby underlining the involvement of the eCB system in social rejection (Schneider et al., 2016). A rapid loss of amygdalar AEA signaling, possibly through a rapid hydrolysis by FAAH, is involved in the reactivity toward different stressors (Hill and Tasker, 2012) hence, enhanced CB1R expression and decreased FAAH levels may occur in the inadequate peer-condition as a long-term consequence to counteract the continuous decreased AEA tone. Notably, a recent study reported alterations of peripheral AEA and 2-AG levels in BPD patients, which indicates a modulatory role of the eCB system in this disorder (Schaefer et al., 2014). Since BPD is characterized *inter alia* by a heightened sensitivity to social rejection, disturbances in social skills, altered pain perception and emotional dysregulation (Schmahl et al., 2006; Bungert et al., 2015), these processes may require intact eCB signaling for normal function. Future experiments will be needed to extend our initial findings onto further neuronal systems involved in social abilities and social rejection. Potentially interesting targets in this context are the endogenous opioid and the central oxytocin system, since both systems are known to functionally interact with the eCB system (Cota et al., 2006; Russo et al., 2012), take part in the regulation of social behavior (Insel and Fernald, 2004; MacDonald and Leary, 2005) and have also been implicated in BPD (Stanley and Siever, 2010; Roepke et al., 2012).

In summary, prolonged periods of inadequate peer-interactions, social rejection or ostracism during adolescence have been shown to have profound negative effects on mental and physical health in humans (Lev-Wiesel et al., 2006; Dempsey and Storch, 2008; Copeland et al., 2013). However, the underlying neurobiological mechanisms remain to be characterized. We here report on persistent impairments in social skills, pain sensitivity, and considerable alterations in eCB signaling in adult female rats that were deprived of adequate playful peer-interactions during adolescence which may therefore provide a suitable approach to model social peer-rejection in laboratory rodents. Our refined inadequate rearing model may also have the potential for a more broadened use in investigating basic behavioral and neurobiological consequences of modulations of social play, a key component of early life social experiences and social development. Finally, the present data contribute to a better understanding of the neurobiological underpinnings of social rejection and, thus increase our insights in the neuropathology of deficiencies in social competence and psychosocial functioning in neuropsychiatric disorders.

## Author Contributions

PS and MS designed the study and analyzed the behavioral data. PS performed experimental work and statistical analysis. PS and LB performed molecular analyses. PS, MS and RS wrote the manuscript. BL, CS, MB and AM-L were involved in the conception and critical revision of the work. All authors contributed to and have approved the final manuscript.

## Funding and Disclosure

This work was supported by the Deutsche Forschungsgemeinschaft (DFG) FOR926, (SP9 (SCHN958/4-1) to MS; to central project CP1 BL) and KFO256 (SCHN958/5-1; IP7; to MS and RS), and AERIAL (BMBF01EE1406C; to RS); the funding entities had no further role in study design; in the collection, analysis and interpretation of data; in the writing of the report; and in the decision to submit the article for publication.

## Conflict of Interest Statement

The authors declare that the research was conducted in the absence of any commercial or financial relationships that could be construed as a potential conflict of interest.
